# Epigenetic Mechanisms of Integrative Medicine

**DOI:** 10.1155/2017/4365429

**Published:** 2017-02-21

**Authors:** Riya R. Kanherkar, Susan E. Stair, Naina Bhatia-Dey, Paul J. Mills, Deepak Chopra, Antonei B. Csoka

**Affiliations:** ^1^Epigenetics Laboratory, Department of Anatomy, Howard University, 520 W St. NW, Washington, DC 20059, USA; ^2^Vision Genomics, LLC, 5725 North Capitol St. NE, Washington, DC 20011, USA; ^3^Department of Family Medicine and Public Health, University of California, San Diego, 9500 Gilman Drive-0628, San Diego, CA 92093, USA; ^4^The Chopra Foundation, 2013 Costa Del Mar, Carlsbad, CA 92009, USA

## Abstract

Since time immemorial humans have utilized natural products and therapies for their healing properties. Even now, in the age of genomics and on the cusp of regenerative medicine, the use of complementary and alternative medicine (CAM) approaches represents a popular branch of health care. Furthermore, there is a trend towards a unified medical philosophy referred to as Integrative Medicine (IM) that represents the convergence of CAM and conventional medicine. The IM model not only considers the holistic perspective of the physiological components of the individual, but also includes psychological and mind-body aspects. Justification for and validation of such a whole-systems approach is in part dependent upon identification of the functional pathways governing healing, and new data is revealing relationships between therapies and biochemical effects that have long defied explanation. We review this data and propose a unifying theme: IM's ability to affect healing is due at least in part to epigenetic mechanisms. This hypothesis is based on a mounting body of evidence that demonstrates a correlation between the physical and mental effects of IM and modulation of gene expression and epigenetic state. Emphasis on mapping, deciphering, and optimizing these effects will facilitate therapeutic delivery and create further benefits.

## 1. Introduction

Western medicine, while excelling at acute care and surgery, puts great emphasis on the chronic use of drugs to suppress the symptoms of illnesses. What is forgotten is that our bodies have a natural wisdom and intelligence; they have an intrinsic knowledge of how to grow, heal, maintain balance, restore homeostasis, and regenerate. Our bodies have evolved over aeons with these capabilities, but when they are suppressed, for example, when nutrition, exercise, and diet are not given adequate attention, or people ingest toxins, then “lifestyle-related” diseases including obesity, diabetes, cancer, and heart disease are much more likely to arise.

Conversely, there is a growing trend by health practitioners to embrace medical philosophies that typically fall outside the parameters normally associated with mainstream medicine. The National Center for Complementary and Integrative Health (NCCIH) has developed a framework for research in this field and has constructed a classification system to standardize the nomenclature [1]. The use of purely nonmainstream healing alone is referred to as alternative medicine, but when used to supplement or “complement” conventional medicine, it is referred to as complementary medicine. Collectively the two are known as complementary and alternative medicine (CAM).

At present the NCCIH recognizes three types of CAM.

*(1) Natural Products.* This category includes the use of herbs, vitamins, and supplements.

*(2) Mind and Body Practices.* This category includes yoga, chiropractic and osteopathic manipulation, meditation, massage therapy, acupuncture, relaxation techniques (such as breathing exercises, guided imagery, and progressive muscle relaxation), Tai Chi, Qi Gong, healing touch, hypnotherapy, and movement therapies (such as the Feldenkrais method, Alexander technique, and Pilates).

**Figure 1:**
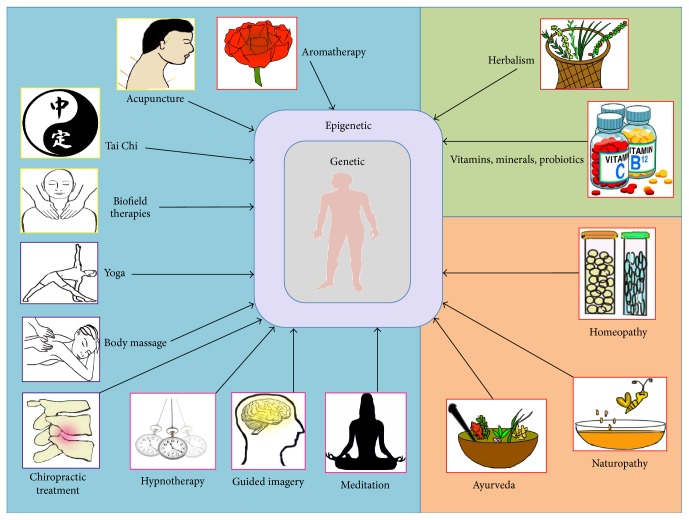
Different types of WSM and IM work through epigenetic modulation. The figure lists prevalent IM practices that have been commonly used to improve health and ameliorate pathological conditions. Since ancient times human civilizations have made use of various natural resources to restore health. It is only in recent years that we have focused on unraveling the underlying mechanism in the light of molecular genetics and biochemistry. NCCIH guidelines classify the different types of IM practices under natural products, mind and body practices, and other practices that are highlighted by light green, light blue, and light orange background colors, respectively. Additional classification of IM practices as material, energy, temporal, and spatial medicine is represented by red, yellow, pink, and purple colored boxes around each graphical icon of IM practices. However, some IM practices can fall under two categories, for example, homeopathy is material and energy medicine. It is interesting to note that mechanisms as widely different as aromatherapy and chiropractic treatment impart psychological impacts prior to their influence on epigenomic DNA in individual cells. The prime impact is always “symptomatic relief” regardless of the practice; however, the pathways leading to that relief are variable, with potential for additional investigative approaches and novel findings. Despite the central dogma that time is the best healer, human civilizations have continually explored modes and means of healing injuries and diseases. In addition to ameliorating the symptoms, these approaches have continued to affect mind, body, and epigenome of somatic cells in the human body. The impacts of some pathways have been partially characterized, while the remainders are being explored from various angles.

*(3) Other Complementary Health Approaches.* This category includes health approaches such as traditional healing, Ayurvedic medicine, Traditional Chinese Medicine (TCM), homeopathy, and naturopathy (Figure 1).

 Different classification schemes, such as those corresponding to the four fundamental elements universal to human experience, matter (material), time (temporal), energy (immaterial), and space (spatial), have also been proposed [2].* Material* medicine is based on principles of biochemistry (e.g., Ayurveda);* temporal* medicine relies on the ability of the brain to focus on past, present, or future contexts (e.g., hypnotherapy);* energy* medicine employs an emphasis on energy flow and meridians (e.g., acupuncture); and* spatial* medicine deals with rearrangement of the body (joints or tissue) in space (e.g., chiropractic treatment). Some of these classifications overlap with the NCCIH ones, as illustrated in Figure 1. However, for the purposes of this review we will adhere to the NCCIH classifications.

A newer NCCIH-defined approach, Integrative Medicine (IM), involves bringing CAM approaches together in an even more coordinated fashion with conventional medicine. IM seeks to restore and maintain health by understanding the patient's unique set of circumstances and addressing their full range of physical, psychological, social, environmental, and spiritual influences. Through such “whole-systems” care, IM goes beyond the treatment of symptoms to address all the possible causes of an illness. Thereby, the patient's immediate health needs as well as the effects of the long-term interchange between biological, behavioral, psychosocial, and environmental influences are considered. The capacity for IM to acknowledge such a unified approach will facilitate the transition from viewing disease as a function of localized afflictions to one occurring on a systemic level. This in turn will allow treatments to expand from narrow, localized interventions to broader, holistic programs.

The “whole-systems” philosophy of IM includes diverse therapies that employ techniques directed towards treating the body and mind in a cohesive, holistic manner [3–9]. Most of these treatment modalities were derived from traditional approaches that viewed the body as a single unit. The list is extensive and includes Ayurveda and Traditional Chinese Medicine (TCM) [10–14]. Increasingly, the value inherent in such practices is being recognized and used in place of standard medical treatment, or in conjunction with it, forming a symbiotic approach. Currently, almost forty percent of adults in the US report using some form of IM with standard medical treatment [15]. Although diverse in style, these practices share a common strategy of stimulating endogenous healing to restore homeostasis and create overall wellbeing.

While practitioners of IM have long advocated the use of traditional therapies, the underlying mechanisms have been mysterious because of limited empirical evidence. In this review, our aim is to elucidate a unifying theme; specifically, we propose that a major part of this mechanism involves epigenetics: the reversible modulation of gene expression through the activity of exogenous or endogenous factors without direct alteration of the genetic code. Over the last decade, the concept of a “fluid” or “plastic” epigenome has evolved because of observations that environmental factors influence the individual and directly impact health status [16].

More recently, research indicates that thought and mental states are capable of affecting gene expression in various ways. Although the exact processes by which mind, thought, and consciousness arise within the brain have yet to be defined, a recent study demonstrated how a synthetic mind-controlled transgene expression device enabled human brain activities and mental states (captured by an EEG headset) to regulate wireless optogenetic implants that radiated infrared frequency and ultimately programmed transgene expression in human designer cells implanted both in mice and in a semipermeable cultivation chamber [17]. In other studies, it has been shown that the autonomic nervous system (ANS), which is generally regarded as a system that cannot be voluntarily influenced, can in fact be brought under some conscious level of control. Results from a case study on a Dutch individual suggested that he could voluntarily activate his ANS through a self-developed method involving meditation, exposure to cold temperatures, and breathing techniques [18]. This example of IM resulted in increased catecholamine and cortisol release as well as a much milder innate immune response during experimental endotoxemia compared with more than 100 subjects who previously underwent the same endotoxemia. In a follow-up study, the effects of his training program on healthy volunteers during endotoxemia showed that the ANS and immune system can indeed be voluntarily influenced. Healthy volunteers practicing the techniques exhibited profound increases in the release of epinephrine, which led to increased production of anti-inflammatory mediators and subsequent dampening of the proinflammatory cytokine response elicited by intravenous administration of bacterial endotoxin [19]. Another “mind over gene” experiment revealed how mental exercise and meditation positively influence telomerase activity in subjects experiencing neuroticism, and these activities prevent the type of telomere degradation typically associated with poor prognosis in breast cancer [20, 21]. Taken together, these and other studies covered in this review suggest that IM methods such as meditation not only reprogram mental attitude and awareness, but do so through epigenetic reorganization and altered gene expression. Collectively, this paper postulates that mentally and materially oriented stimuli delivered through IM can switch genes on or off, presenting a new paradigm of how IM functions.

## 2. Presentation of the Hypothesis

The hypothesis presented in this review is that IM operates through the environment-body and mind-body interfaces via engagement of the epigenome [22]. It is effective not just in treating illness, but also in alleviating pain and stress [23]. Epigenetic mechanisms include chromatin folding and attachment to the nuclear matrix, packaging of DNA, covalent modifications of histone tails (acetylation, methylation, and phosphorylation), and DNA methylation, which taken together provide a plausible explanation for nongenetic disease transmission [24, 25]. External parameters such as diet and exercise represent prominent elements in the induction of such epigenetic changes, resulting in health benefits through genomic regulation [26, 27]. Currently, nutritional compounds and phytochemicals are amongst the most potent known regulators of epigenetic function. Not only are epigenetic factors like diet potent modulators of gene expression, but this modulation can be reversible and/or heritable [28]. Control is exerted through cellular signaling pathways that eventually affect physiological and developmental events [29, 30].

Emerging epigenetic data indicate that IM techniques are fully viable and credible approaches for treating illness, on par with Western medical counterparts in terms of goals and risks [31]. In fact, these approaches have proven superior to some conventional treatments because they tend not to elicit such serious side effects. Since it is understood that epigenetic factors are potent modulators of gene expression and that such modulations can be reversible, it is logical to presume that factors and practices within the IM purview are capable of evoking an epigenetic response. IM practices including Ayurveda, Tai Chi, yoga, and Reiki may alter gene transcription and cause modifications at the level of DNA methylation and histone methylation and/or acetylation [32]. Further, neural circuits are plastic and the effects of such therapy may result from epigenetic changes at the neurobiological level. For example, transcriptional analysis of depression-associated behavior syndromes like postpartum depression that involve a differential immune activation and a decreased transcriptional engagement in cell proliferation, DNA replication, and repair processes might provide markers that can be targeted through the epigenetic mechanisms of IM therapies. While the current scientific literature does not specifically address questions related to mechanism or potential side effects of IM, it does discuss beneficial results and reveals the promise of a nonpharmacological alternative to healing through a safer, less invasive format [33]. Thus, IM often prevails over standard Western options from a cost/benefit standpoint.

In addition, mind-body medicine techniques such as yoga and meditation involve a crucial psychological factor that can help in the management and treatment of an array of diseases with a genetic or behavioral/neurobiological basis, with their power in alleviating pain related symptoms associated with chronic conditions like arthritis [34].

### 2.1. Proposed Epigenetic Mechanism for Complementary, Alternative, and Integrative Medicine

Epigenetic modifications such as DNA methylation and histone modification can be regulated by external environmental factors in addition to the inherited genetic profile of an individual. Such modifications are deterministic of disease onset from childhood through adulthood [29]. The resulting architecture of the epigenome (i.e., the specific pattern of epigenetic signatures including DNA methylation and histone modifications throughout the genome) is responsible for the physiological processes and psychological states inherent within everyone. Bearing this in mind, we propose that various types of IM function as positive epigenetic factors.

**Figure 2:**
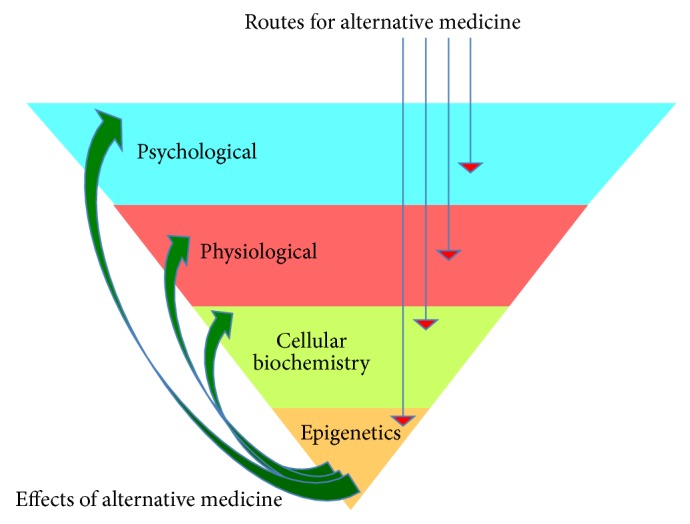
IM: routes and effects. The figure represents how IM works at different levels such as the psychological, physiological, biochemical, and/or epigenetic levels. The red arrows indicate the different initial levels at which IM can exert its action so that the final action is exerted at the epigenome. An altered epigenome then produces beneficial effects at different levels indicated by green arrows. An IM approach can work at these different levels through a specific hierarchical sequence, or at different levels simultaneously. Some approaches can first act directly at the epigenetic level, surpassing the psychological and physiological levels, for example, the use of curcumin (material medicine) to treat cancer. Conversely, some IM approaches might first act at the psychological level and work their way “downwards” into the epigenome. For example, Ayurveda can act directly on the epigenome, whereas meditation works at the psychological level first. Yoga is an example of a modality that operates at the psychological and physiological levels simultaneously, ultimately altering the epigenome. In certain disease conditions, it may be required that the approach must operate directly at the epigenetic level first. For example, in illnesses where the approach has no physiological or psychological effects, a patient might have their chromatin locked in such a way that is very difficult to alter through a psychological → physiological → epigenetics cascade. For example, in the case of a “psychological block,” it would be first necessary to alter and increase the epigenetic plasticity before changing the psychological state. After the alteration of the epigenetic profile, the effect can manifest in the form of gene expression and biochemical changes, physiological and/or psychological changes. Note also that the DNA sequence (genetic layer) of an individual can also modify the degree of response to any given IM approach. Different types of combination therapy can be used for treatment of severe illnesses.

It is also important to understand that IM has the capacity to work at different levels, psychologically, physiologically, and/or directly at the level of the epigenome in the nucleus of a cell (Figure 2). Moreover, some IM approaches might first act at the psychological level, eventually working their way “downwards” into the epigenome in a “domino fashion.” Conversely, other IM practices may directly impact the epigenome first, surpassing altogether the psychological and physiological levels and then working their way “upwards” towards these levels. For example, meditation likely works primarily at the psychological level first, whereas yoga works at the psychological and physiological levels simultaneously, before altering the epigenome. Thus, the myriad effects of IM are visible from the level of gene expression to the overall physiology and psychology of the patient (Figure 2). Furthermore, the actual DNA sequence of an individual can modify the degree of response to an alternative medicine approach (Figure 1).

In the case of certain diseases or degenerative maladies, it may be mandatory for the IM approach to directly target the epigenome first. For example, in severe illnesses such as schizophrenia, a therapeutic strategy that targets psychological and physiological response prior to affecting the epigenome may prove ineffective in function or duration. For example, the chromatin may be locked (tightly wrapped) in such a manner that renders it difficult to alter through this cascade (i.e., psychological → physiological → epigenetic). Hence, for such diseases an IM approach capable of directly altering the epigenome first by initially circumventing/bypassing the psychological and physiological levels might be considered a more effective strategy.

### 2.2. Direct and Indirect Epigenetic Pathways

**Figure 3:**
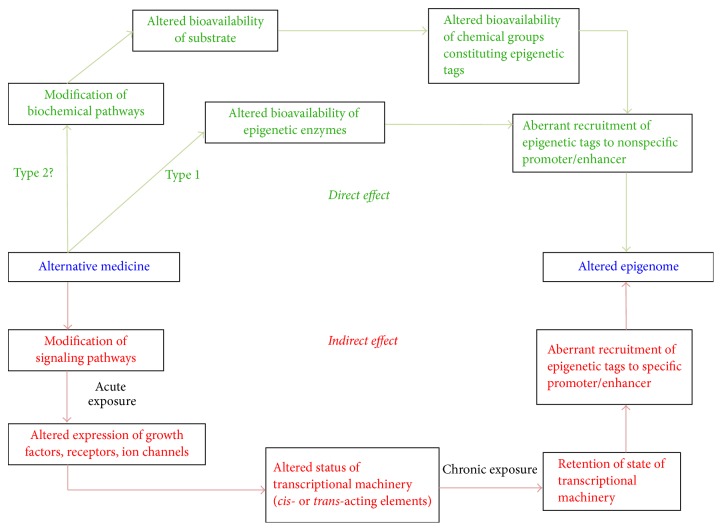
Overview of the proposed epigenetic mechanism through which IM modulates healing. The figure represents two different routes through which IM can modify the epigenome leading to altered gene expression. Effects exerted by IM can lead to direct and indirect effects. Green represents direct effects and red represents indirect effects. The direct pathway can operate in two different ways, namely, Type 1 and Type 2. In Type 1 direct pathway, IM directly exerts an effect on the epigenetic enzymes such as DNMTs, HDACs, HATs, HMTs, and HDMs, such that there is an altered bioavailability of these enzymes in the cell. A Type 2 direct effect is when IM interferes with a biochemical pathway such that there is altered availability of a metabolite required for constituting an epigenetic tag. Both cases can result in beneficial recruitment of epigenetic tags to promoters, ultimately establishing an altered epigenetic profile. In the indirect pathway, IM indirectly exerts an effect on the epigenome by first interfering with signaling pathways in the cell. An acute exposure to the IM modality can cause altered expression of growth factors, receptors, ion channels, and so on resulting in nonhomeostatic cellular processes. This in turn might lead to an altered status of transcriptional machinery (bound or unbound to the promoter/enhancer) and its bioavailability in a cell. A chronic exposure to the IM modality might lead to retention of such state of transcriptional machinery (bound or unbound to the promoter/enhancer) causing altered gene expression as well as beneficial recruitment of epigenetic enzymes, leading to permanent addition or removal of epigenetic tags to specific promoter/enhancers. This consequently establishes an altered, preferable “healed” epigenetic profile.

**Figure 4:**
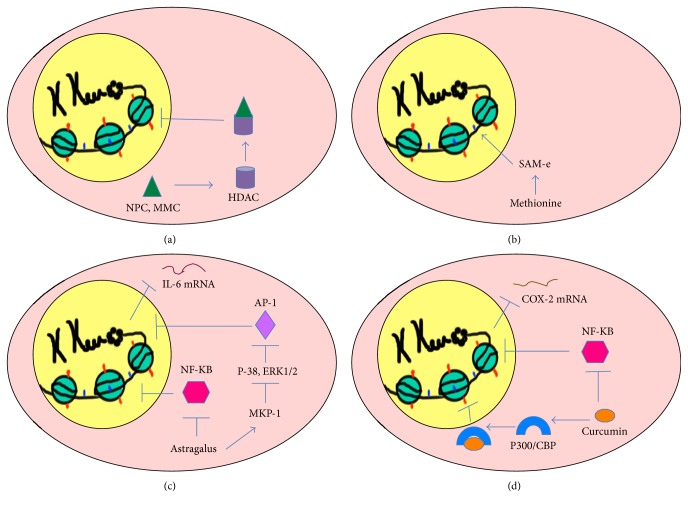
Direct, indirect, and combined epigenetic pathways of Integrative Medicine. The figure represents a summary of the epigenetic mechanisms underlying different IM approaches. The cell is represented as oval pink structure with a yellow nucleus. The fine structure of chromatin comprised of DNA wrapped around histones in the nucleus is the ultimate regulatory component through which IM approaches manifest their outcomes. Red marks on histones represent acetyl groups on histone tails (histone acetylation) and blue marks represent methyl groups on DNA (DNA methylation). (a) Type 1 direct pathway: Traditional Chinese Medicine. Traditional Chinese Medicine (TCM) compounds like Ningposides C (NPC) and Monomethylcurcumin (MMC) (represented by green triangles) act as HDAC2 (represented by purple cylinder) inhibitors that inhibit deacetylation of histones and relax the chromatin structure. The mode of action of NPC and MMC is through a Type 1 direct pathway since they directly interfere with the epigenetic enzyme HDAC. (b) Type 2 direct pathway: herbal methionine. Dietary compounds like herbal methionine (HM) that donate methyl groups and increase SAMe levels in the body are important regulators of nuclear DNA methylation. An increased production of SAMe from HM increases the bioavailability of methyl groups (metabolite) that contribute to the constitution of epigenetic tags, specifically affecting DNA methylation levels. Thus, HM follows the Type 2 direct pathway by interfering with the bioavailability of compounds that constitute epigenetic tags. (c) Indirect pathway: Astragalus. Astragalus extract has anti-inflammatory properties and can promote its effects through two different pathways. Firstly, it inhibits p38 MAPK and ERK1/2 via stimulation of MPK that in turn blocks the nuclear translocation of AP-1 (lavender diamond) responsible for expression of proinflammatory cytokine IL-6. Secondly, it interferes with the nuclear translocation of NF-*κ*B (pink hexagon) and inhibits NF-*κ*B-mediated transcription that in turn activates proinflammatory genes. Thus, Astragalus follows an indirect epigenetic pathway by interfering with cellular signaling pathways. (d) Combined pathway: curcumin. Curcumin (orange oval) possesses anti-inflammatory activity that operates through a combination of direct and indirect pathways. Through the direct epigenetic pathway, it specifically inhibits a specific p300/CBP HAT (blue semicircle) thereby reducing histone acetylation and through the indirect epigenetic pathway it blocks pathways involving the transcription factor NF-*κ*B (pink hexagon) that in turn block the production of COX-2. Thus, curcumin operates through a combination of direct and indirect pathways.

Our working hypothesis is that IM operates through two independent epigenetic pathways, namely, direct and indirect, or through a combination of the two (Figures 3 and 4). We have previously described a model elucidating the detailed mechanism underlying epigenetic pathways [26]. Here we propose that any form of IM operates through one or both of these types of epigenetic pathways that ultimately modify the epigenome and result in altered gene expression and/or psychological and physical states.

The direct epigenetic pathway can be divided into two types: Type 1 and Type 2 (Figures 3 and 4). Any type of IM approach that directly exerts an effect on epigenetic enzymes such as DNA methyltransferases (DNMTs), histone deacetylases (HDACs), histone acetyltransferases (HATs), histone methyltransferases (HMTs), and histone demethylases (HDMs) such that there is an altered bioavailability of these enzymes in the cell is said to follow Type 1 direct pathway. Alternatively, when an IM approach interferes with the operation of a biochemical pathway(s) such that there is altered bioavailability of a metabolite constituting an epigenetic tag, it is said to follow Type 2 direct pathway. Types 1 and 2 direct pathways can alter recruitment of epigenetic tags to nonspecific promoters resulting in the possibility of genome-wide changes in gene expression (Figures 3 and 4).

For example, of 3,294 Traditional Chinese Medicine (TCM) compounds evaluated to date, 1,170 (36%) have been found to interact with human histone-modifying enzymes [35]. Of those, TCM compounds such as Ningposides C and Monomethylcurcumin act as HDAC2 inhibitors [36] and constitute examples of Type 1 direct pathway modulators (Figure 4).

Conversely, dietary compounds containing methyl group donors are important regulators of nuclear DNA methylation [37]. S-Adenosylmethionine (SAMe) is the main methyl donor in various methyltransferase reactions [38] and is produced in the body from methionine [39, 40]. Methionine is the active component of herbal methionine (a combination of herbs* Cicer arietinum*,* Triticum sativum*,* Phaseolus mungo*,* Mucuna pruriens*, and* Allium cepa*) that is used in Ayurveda and herbalism [41]. Amongst these herbs,* Mucuna pruriens* is an antioxidant used in Ayurvedic medicine to treat Parkinson's disease [42].* Mucuna pruriens* extract is high in methionine and capable of affecting SAMe levels in the body. An increased production of SAMe from herbal methionine (HM) increases the bioavailability of methyl groups (metabolite) that constitute epigenetic tags (in this case DNA methylation). Thus, the Ayurvedic approach involving IM follows Type 2 direct pathway modulation (Figure 4).

The alternate epigenetic pathway is capable of indirectly exerting an effect on the epigenome through initial interference with an active or inactive signaling pathway in the cell [26]. Acute exposure to certain IM approaches may lead to an altered expression of growth factors, receptors, or ion channels disrupting homeostatic cellular processes. This in turn could alter the status of transcriptional machinery (bound or unbound to the promoter/enhancer) as well as its bioavailability within a cell. On the other hand, a chronic exposure to the IM approach might result in retention of a transformed state of transcriptional machinery (bound or unbound to the promoter/enhancer). These indirect pathways can be further divided into 2 subtypes:* cis*-acting and* trans*-acting. A signaling molecule or regulatory protein when bound far from the promoter and transcription start site (TSS) is* trans*-acting and does not have a strong influence on the promoter. Conversely, the* cis*-acting element is bound close to the promoter and directly to the DNA sequence including the TSS, facilitating a strong enough influence over the promoter to induce or upregulate transcription. This could alter gene expression and/or cause abnormal binding of epigenetic enzymes, making permanent additions or removal of epigenetic tags to specific promoter/enhancers. Thus, IM approaches resulting in such indirect pathway activity could alter the epigenome and gene expression, such that their effects could be extrapolated as positive changes in psychological and physiological states (Figure 2).

An example of an IM approach utilizing the indirect pathway is the TCM called Astragalus (*Astragali radix*), used for treating inflammation (Figure 4). Astragalus extract has anti-inflammatory properties that stimulate inflammatory genes, leading to reduced expression of iNOS, COX-1, COX-2, IL-6, IL-1beta, and TNF-alpha [43, 44].* Astragali radix* stimulates mitogen-activated protein kinase phosphatase 1 (MKP-1), leading to inactivation of p38 and ERK 1/2 and ultimately to reduction of inflammation [43]. The mechanism behind this involves the negative regulation of p38 (a mitogen-activated protein kinase) and Extracellular-signal Regulated Kinase (ERK) pathways by MKP. When otherwise upregulated, the p38 and ERK pathways enhance expression of proinflammatory cytokines such as IL-6, IL-8, and COX-2 [45, 46]. In addition,* Astragali radix* interferes with the translocation of NF-*κ*B to the nucleus, possibly through allosteric regulation, and inhibits NF-*κ*B-mediated transcription that can otherwise promote inflammation [43]. These actions explain the anti-inflammatory activity of Astragalus and are based on an indirect epigenetic pathway that can interfere with other cellular signaling pathways (Figure 4).

As a final example, curcumin, long used in Ayurvedic and TCM as an anti-inflammatory agent, operates through a combination of direct and indirect pathways [26] (Figure 4). Curcumin inhibits a specific HAT, eventually reducing histone acetylation (affecting nonspecific promoters: Type 1 direct effect) and/or interfering with signaling pathways involving the transcription factor NF-*κ*B leading to decreased production of inflammatory COX-2 (affecting specific promoters: Type II indirect effect) [47, 48]. Therefore, this example of IM involving curcumin operates through a combination of direct and indirect pathways (Figure 4).

## 3. Explication of the Hypothesis

The postulation that an epigenetic mechanism operating through IM at the molecular level can elicit beneficial effects at the organismal level demands a comprehensive discussion and evaluation. Current literature does support this notion and provides some evidence that IM functions at an epigenetic level. However, research efforts going forward must focus on deciphering the cellular responses to different IM practices in conjunction with careful epigenetic profiling of both treatment and control groups to completely decipher and understand all the relevant mechanisms. Only such a concerted effort will ascertain exactly how the benefits of IM stem from epigenetically regulated gene expression. To this end, a detailed compilation of the three NCCIH categories of complementary medicine (natural products, mind and body practices, and other complementary health approaches) will be presented from an epigenetic perspective; taken together, these products and practices are part of the whole-systems approach of IM (please refer to Figure 1 for graphical classification).

### 3.1. Natural Products

IM approaches falling under the category of natural products refer to the use of vitamins and minerals, probiotics, and dietary supplements that are sold over the counter.

#### 3.1.1. Herbalism

Herbalism is the practice of using traditional herbs as medicines in treating health related problems. Bioactive compounds in herbs including polyphenols, isothiocyanates, saponins, and terpenoids, are drug-like and may act in different ways compared to single-target drugs [49]. The classification of herbalism under natural products is ambiguous as it overlaps with the third category of “other complementary health approaches.” Use of botanical herbs can thus come under the third category as it may apply to traditional herbalist practices such as TCM. A study of TCM, a form of herbalism which evolved over the course of 2,100 years and remains popular with Far East Asian populations, evaluated various TCM medicinals and pharmacological chemicals [50]. It was shown that of the 3,294 TCM herbs evaluated 29.8% of them acted via modulation of the epigenome through interactions with polycomb groups and methyl-CpG binding proteins [50]. Of 200 government-approved TCM herbs, approximately 99% functioned on an epigenetic level, especially through histone modifications [35]. TCM herbs have also been recognized for their chemopreventative potential through epigenetic modifications of cancer stem cells following ingestion of these phytochemicals [51].

Herbalism may also depend on the principles of transgenerational epigenetics, whereby a connection between people and their local environment, including exposure to locally grown herbs, renders them positively receptive to local healing herbs that favor both individual and germline survival [52]. This model suggests that an individual's earliest herbal exposures may program somatic cells for therapeutic herbal receptivity and additionally “prime” the egg cells which generally have long latency (up to 60 years).

### 3.2. Mind and Body Practices

This constitutes the major NCCIH category of complementary medicine approaches involving several popular therapies, movements, and exercises taught by qualified healers.

#### 3.2.1. Acupuncture

The use of acupuncture therapy involves the insertion of needles at different points in the body aimed at pain relief. The acupuncturist restores “Qi” or “Chi” (a hypothetical flow of energy) to deficient areas, rerouting from areas that have excess, to restore equilibrium of energy flow in the body. Acupuncture has been used successfully to treat multidimensional episodes of pain and is recognized as a biopsychosocial therapy for pain management [53]. For example, it has been clinically useful for prevention of myocardial infarction (MI) by strengthening cardiovascular function, effecting angiogenesis, and protecting against further injury resulting from apoptosis of myocardial cells following MI [54]. Interestingly, acupuncture has been shown to upregulate vascular endothelial growth factor (VEGF) expression through direct H3K9 acetylation at the VEGF promoter inducing angiogenesis in rat MI models [55]. Similarly, the mechanism of electroacupuncture used for alleviating symptoms of stable angina pectoris (chest pain) related to myocardial ischemia is modification of gene regulation and remodeling of epigenetic marks including H3K4me1, H3K4me2, and H3K27ac [56]. These findings suggest a link to epigenetic regulation of regeneration and cellular apoptosis during and after MI via acupuncture.

Another study involving the Sirt2 gene (present in human and rat neurons) responsible for deacetylating histones and promoting chromatin compaction demonstrated that acupuncture epigenetically modifies levels of miR-339 that regulate Sirt2, ultimately interfering with the miRNA-339/Sirt2/NF-B/FOXO1 axis [57]. In the case of Parkinson's disease (PD), acupuncture treatment in murine PD models results in mice that display neuroprotective effects through changes in p53 signaling that significantly aid recovery in terms of behavior and molecular composition of neurons [58]. However, not all results have been positive [59]. Studies involving in vitro fertilization have shown acupuncture to produce no increase in the success rate of clinical pregnancy [60].

#### 3.2.2. Meditation

Meditation is a popular practice, in which the meditator aims to achieve a peaceful mind and tranquil thoughts through awakening of the inner consciousness. Enhanced psychoemotional balance and focused attention skills in long-term meditators are linked to the upregulating functional effects seen on frontoparietal attention networks and frontolimbic networks of emotional control, which could be the neurophysiological mechanisms of action for reported psychoemotional and cognitive effects [61]. Experiences of higher states of consciousness through meditation are ultimately associated with changes at the transcriptional level. This is supported by evidence from a study involving whole-genome expression analysis of long-term meditators who possessed distinctive differential gene expression profiles (involving approximately 1,000 genes) compared to controls [62]. It has also been suggested that meditation influences telomere length in a positive way by reducing cognitive stress and stress arousal, increasing positive states of mind, and stimulating hormonal factors that promote telomere maintenance, altogether decelerating cellular senescence [63].

Meditation is often used as a preventative activity against “lifestyle diseases” such as cancer, obesity, and diabetes. For example, a study involving a male population with low-risk prostate cancer that participated in an exclusive, intensive nutrition and lifestyle intervention (meditation, yoga guided imagery with a specially designed diet) showed significant improvements in weight, abdominal obesity, blood pressure, and lipid profile with beneficial changes in gene expression and modulation of signaling pathways [64]. In addition, mind-body therapies have a significant effect on the cellular and genomic markers of inflammation, eliciting decreased expression of inflammation-related genes and reduced signaling through the proinflammatory transcription factor NF-*κ*B [65].

Some studies suggest that this role of consistent meditation in influencing the integrated mind-body envelope or in triggering biological effects involves changes at the epigenetic level [66]. Meditation reduces oxidative stress and biophoton emission (a spontaneous emission of ultra-weak photons emanating from all living systems [bioluminescence] linked to the endogenous production of excited states) that suggests the involvement of alternative metabolic pathways with fewer products of oxidation and reduced free-radical interactions occurring from conformational changes in chromatin [67, 68]. Studies involving profiling of key epigenetic chromatin modulators such as HMT, HDM, HDAC, and DNMT in meditators and controls showed that the meditators displayed rapid peripheral changes in their expression of HDAC2, HDAC3, and HDAC9 along with global histone modifications (H4Ac, H3K4me3) [69]. Meditators also expressed HDAC2 and proinflammatory genes RIPK2 and COX-2 at lower levels, resulting in better cortisol recovery in social stress tests, supporting the therapeutic benefits of meditation in treating social stress disorders [69].

As mentioned earlier, a recent study showed that a synthetic mind-controlled transgene expression device enabled human brain activities and mental states (captured by an EEG headset) to regulate wireless optogenetic implants radiating infrared frequency, ultimately programming transgene expression in human designer cells implanted in mice and in a semipermeable cultivation chamber [17]. It also showed that different brain states achieved during biofeedback control, mental concentration, and meditation can result in differential production of secreted alkaline phosphatase (due to differential gene expression) in designer cells in culture and those implanted in mice [17]. Along similar lines, meditation can be used as a therapy for treating neurodegenerative diseases such as Alzheimer's by preventing cognitive decline [70]. Thus, meditation should be considered as a potential candidate for alteration of gene expression in a safe and beneficial fashion.

#### 3.2.3. Chiropractic

Chiropractic treatment focuses on maladies of the musculoskeletal system and is based on the manipulation of subluxated and misaligned joints, especially the spinal cord, that in turn cause pain in nerves and muscles. A study of treatment of pregnancy-related heartburn showed that chiropractic intervention can be a credible alternative to pharmaceuticals [71]. Additional studies on efficacy of spinal manipulation for the treatment of pregnancy-related low back pain showed an improvement in function and decrease in pain [71]. It is well known that epigenetic influences acting on a pregnant mother also affect the in utero fetus [26]. Therefore, the study suggests a possible role for chiropractic treatment in promotion and enhancement of long-term fetal and infant health benefits through epigenetic pathways. Not only have spinal manipulation techniques been used to relieve symptoms arising from chronic low back pain, but they have also proven effective in treatment of vertebral subluxations in diabetic patients [72, 73]. This research suggests that long-term chiropractic therapy may provide a tangible solution to chronic diseases [74].

#### 3.2.4. Massage

Body massages are known to have a soothing effect on skeletal muscle as well as relieving the pain associated with muscle cramps. Recently, it was shown that regular sessions of sixty-minute massage significantly reduced chronic neck pain, also suggesting that therapeutic massage may represent an efficient means for alleviating chronic body ache [75]. Massage therapy helps in reducing inflammation and hence pain, through the activation of mechanotransduction-related signaling pathways and mitochondrial biogenesis, operations that are necessary for mitigating cellular stress [76]. Quite frequently it has been observed that a good massage induces sleep, thereby relieving stress.

The connection between stress relief and body massage suggests that touch may heal the disturbed psychological status of a stressed individual. This observation can be cross-referenced against studies involving licking or grooming (LG) of pups by mother rats and arched-back nursing. Such behavior resulted in improved hypothalamus-pituitary-adrenal responses to stress in offspring that perpetuated into adulthood via changes in hippocampal glucocorticoid receptor expression through decreased DNA methylation at the gene promoter and increased acetylation of lysine 9 on histone 3 (H3K9) [77, 78]. LG rats not only display less anxiety, but deliver better performance in tests of learning and memory than low LG pups [79]. The studies emphasize the importance of nurturing and care, especially with respect to understanding how a healing touch can enhance mood and improve emotional responses to stressful situations. Hence, the pain relieving, soothing/calming effects of massage correspond to the improved response to stress in LG rats and both cases may have a similar epigenetic basis wherein sensory experiences regulate neuronal plasticity and behavior via epigenetic mechanisms.

In the case of humans, an interesting study that correlated the effects of LG or sensory stimulation observed in rodent offspring to human preterm infants showed that body massage enhanced the maturation of electroencephalographic activity of the brain and of visual function which can be linked to high levels of IGF-1 in their blood (also in the cortex of rat pups) [80]. Therefore, it is likely that the sensory stimulation from massages transforms into epigenetic modulation at a neurobiological level resulting in altered gene expression that triggers a positive neuronal development along with wellness and relaxation.

#### 3.2.5. Yoga

Yoga is a form of mind-body exercise that emphasizes the concept of “a healthy mind leads to a healthy body.” Given the popularity of yoga, there is increasing evidence that this traditional form of exercise invigorates both the mind and body by introducing changes at the psychological level with benefits including reduced risk of cardiovascular disease and improvements in sleep, mood, perceived stress, and blood pressure [81, 82], plausibly via epigenetic mechanisms. Yoga practitioners might develop autonomic responses to stress along with self-regulating coping behaviors that may help in changing the perception of life-stressors. Such responses might occur through positive influence on the person's physiological reactions to their life situations that originate via both top-down (psychological reappraisal) and bottom-up influences (responses to autonomic changes such as reduced inflammation or enhanced vagal tone) on prefrontal cortex subregions that can control allostatic load [83].

Yoga is an excellent alternative therapeutic approach for treating “lifestyle problems” caused by epigenetic or environmental factors such as mood and anxiety disorders [84]. Records indicate that yoga improves the daily dietary choices made by practitioners, including avoidance of high-fat foods and increased intake of fresh vegetables and whole grains; this in turn lowers the risk for cardiovascular disease and presents a positive alternative for combatting the obesity epidemic and eating disorders [85].

Yoga and related practices induce rapid changes in the genetic expression of peripheral blood mononuclear cells within two hours as compared to controls, suggesting a physiological change involving immune-related cells [86]. Yoga along with meditation has been proposed as a dual therapy for improving the chances of smoking cessation and could represent a potential treatment for drug addiction [87]. Yoga-mediated reduction of hypertension has long been investigated and has recently been reported to reduce blood pressure in hypertensive patients [88]. In a promoter-based bioinformatics analysis study it was shown that yogic meditation techniques followed by family dementia caregivers trigger gene expression changes, conducive to stress reduction [89]. Such changes in stress reduction are primarily based on reduced activity of proinflammatory NF-*κ*B family of transcription factors and increased activity of Interferon Response Factors that are related to transcription of innate antiviral response genes [89].

A variation of yoga known as Bikram Yoga (or hot yoga) uses a room heated to 40°C with 40% humidity, beneficial for strength conditioning and flexibility. This practice has been shown to increase overall levels of mindfulness in participants, along with reduction in the level of perceived stress [90]. One study mentions the role of Bikram Yoga on the shorter duration of transitioning into sleep after nocturnal awakening, a possible treatment for insomnia [91].

Altogether, yoga increases muscular strength and flexibility, enhances cardiovascular and respiratory functions, aids in addiction recovery, keeps a check on anxiety and depression, treats chronic pain, improves circadian rhythms, and promotes wellness and improved life quality [92]. Yoga along with other lifestyle changes including diet has been shown to enhance telomere length and telomerase activity [93, 94]. Since telomeres are epigenetically regulated and their damage leads to senescence via epigenetic mechanisms [95, 96], it seems safe to speculate that the positive health effects and antiaging benefits of yoga occur through epigenetic regulation acting to maintain and/or extend telomere length.

#### 3.2.6. Hypnotherapy

Hypnotism is a form of psychotherapy that aims to induce a sleep-like state, causing unconscious or subconscious changes in the subject that result in heightened suggestibility and responsiveness. The goal is to allow the subject to form new responses, behaviors, or attitudes to improve quality of life and mental outlook. Hypnotherapists successfully employ this method to treat psychological disorders. Hypnotherapy can also be used for reducing pain associated with fractures, burns, and lacerations and for analgesic purposes during painful procedures including needle sticks, laceration repair, and obstetric/gynecologic problems [97]. It can facilitate reduction of acute anxiety, increase cooperativeness in children for certain procedures, and promote the diagnosis and treatment of acute psychiatric conditions [97]. One study showed that an ideoplastic (intuitive creativity) process of hypnotherapy might involve an upregulation of genes necessary for stem cell growth as well as a reduction in cellular oxidative stress and chronic-inflammation to change cognitive behavior [98, 99]. Such altered gene expression may result from changes in the DNA methylation pattern affecting the brain with respect to mental health and mood, which are reversible [98]. A hypnotherapy-based clinical study showed improvement in symptoms, reduced dependence on medication, and an 80% improvement in quality of life in participating patients suffering from irritable bowel syndrome (IBS) [100]. Self-hypnosis when combined with asthma management for serious medical and psychological conditions such as associated panic attacks showed an improvement in the ability to reduce dependency on bronchodilators amongst patients [101].

#### 3.2.7. Guided Imagery

Guided imagery is a practice that primarily recruits the mental capabilities of an individual to relieve conditions ranging from stress and anxiety to headaches and nausea, and most techniques also include a mild form of self-hypnosis. It relies on input from all the senses to create the image of a desired situation in the mind, such that the experience has a powerful impact on healing the mind and body comprehensively. Guided imagery has been known to help patients deal with psychological issues and is a common practice amongst cancer patients. It can also induce a state of happiness in demoralized individuals [102] and may prove useful in treating posttraumatic stress disorder (PTSD) plaguing thousands of veterans. A study with active duty military personnel that participated in guided imagery therapy showed a significant reduction in PTSD and related symptoms after attending 6 sessions over 3 weeks [103]. Use of guided imagery for young children suffering from sickle cell disease resulted in an increase in self-efficacy and reduction in pain intensity along with reduced dependence on analgesics, which might be due to an epigenetic alteration of pain sensitivity-related gene expression [104].

#### 3.2.8. Psychoeducation

Psychoeducational training strives to improve the mental health of patients suffering from disorders such as depression, schizophrenia, and other psychotic or personality disorders, has yielded many promising results [105], and may also work at the epigenetic level [106]. It has also been suggested that psychoeducational intervention may be developed to combat physical pain, such as that experienced by cancer patients [107]. Patients suffering from IBS showed improvement with respect to gastrointestinal symptom severity, visceral sensitivity, and depression following a psychoeducational group intervention [108]. In the case of schizophrenia, a novel technology-based approach called the Health Technology Program prevents symptom relapses and rehospitalizations by managing psychosis through direct treatment at patients' homes [109]. This approach provides in-person relapse prevention planning utilizing technology-based treatment specifically designed to incorporate cognitive-behavioral therapy for psychosis, family psychoeducation for schizophrenia, and prescriber decision support through a Web-based program. Such technology-based programs are delivered through smartphones and computers, making them cost-effective in administration of flexible, personalized evidence-based treatments [109]. These findings indicate that alterations in cognition and fear within patients seem to operate by inducing an epigenetic change involving the neural circuitry.

In addition, psychoeducation can benefit the patient through the phenomenon of emotional contagion. Individuals are capable of mimicking facial, vocal, and postural expressions and thus may feel the emotions of other individuals [110]. Emotional states can be transferred between individuals, causing them to experience the same emotions without any awareness of this phenomenon [111]. For example, an individual's happiness is dependent upon the happiness of those with whom they share close connections [112]. Also, it has been noted that female friends are highly influential in the spread of depression from one individual to another [113]. These are all examples of emotional contagion, and such instances have also been recorded in a large-scale network. It has been shown that emotions expressed by people via posts on social networks, such as Facebook, can influence the public at large who are participating on the same social media sites [111]. This type of emotional contagion excludes direct interaction between individuals and operates in the complete absence of nonverbal cues [111].

#### 3.2.9. Aromatherapy

The use of essences and fragrances harvested from essential oils in treating psychological disorders has recently gained popularity, yet the underlying mechanism remains elusive [114]. This kind of approach for treating psychological disorders is based on the premise that the inhalation of certain fragrances tends to trigger an olfactory response, which in turn results in release of neurotransmitters such as dopamine that can regulate mood and behavior. It is suspected that such effects may have an epigenetic basis [114]. Studies show that aromatherapy massage proves to be more effective than regular massage therapy in relieving menopause-related psychological symptoms in pre- and postapplication intervention groups in Iranian women as compared to controls [115]. While the dynamics of menopause and menopausal symptoms are governed by the epigenetic regulation of certain genes [26], it is likely that aromatherapy might be targeting the epigenetic signature to relieve menopausal symptoms. In an interesting study, rats stressed from sleep deprivation were exposed to the aroma of roasted coffee beans and it was found that several genes responsive to coffee aroma or stress were differentially expressed compared to controls [116]. Upregulation of genes such as nerve growth factor receptor suggests an antioxidant activity; glucocorticoid-induced receptor gene upregulation suggests anxiolytic effects [116], and so forth. These results indicate that the stress-relieving properties of coffee aroma may operate through epigenetic mechanisms.

#### 3.2.10. Biofield Therapies

Energy medicine comprises numerous biofield therapies, complementary medicine modalities that remain controversial, and involves manipulation of a putative “biofield” [117], including modalities such as healing touch, therapeutic touch, and Reiki. The clinical evidence for biofield therapies suggests that they are effective in symptom management for pain and cancer, along with diseases like arthritis, dementia, and heart disease [118]. In terms of the capacity to objectively assess the hypothetical biofield, some investigators hypothesize that biophoton emission [119] provides potential insight into aspects of biofield activity; but currently, there are no instruments to reliably measure or validate this claim [120]. Biophoton emission-based cell-to-cell signaling operates through a subtle body of light that regulates the physical body, such that coherent biophoton signaling can regulate functions including cell-cell orientation detection, neurotransmitter release, and long-range interactions observed for leukocyte respiration [120].

Reiki is another type of energy therapy that involves laying on of hands to scan blocked energy from negative patterns that prevent healing. In other words, Reiki deals with energy fields, biofields, and energy flow. Reiki potentially involves a cell-to-brain connection that is capable of changing brain states and influencing physiological processes. Of note is that Merkel cells, located in the epidermis of the palms and fingers, are excitable cells that are in close contact with sensory nerve endings and contain melanosomes responsible for human magnetoreception [121]. A brain-to-Merkel cell connection is bidirectional, and the ability of the multisensorial Merkel cells to absorb and radiate electromagnetic frequencies may be the source of Reiki's efficacy [121]. Given that the brain is a malleable organ (in terms of programming) and that the neuromotor patterns are changeable, such biofield modes of treatment may help relieve deep emotional past-patterns and usher in the positive energy necessary to facilitate healing. The recruitment of energy as the basis of healing in modalities such as Reiki, in conjunction with the underlying plasticity of the brain, suggests that their ability to heal depends on an epigenetic mechanism.

#### 3.2.11. Tai Chi

Tai Chi is an ancient Chinese mind-body exercise technique that includes movements combining deep-breathing, relaxation, and meditation. In addition to falling into the spatial medicine category, movement therapies such as Tai Chi and Qi Gong also fall into the energy medicine domain. A study involving the DNA methylation profiles of female Tai Chi practitioners has shown beneficial epigenetic changes related to six CpG dinucleotide marks, of which four exhibit demethylation and two exhibit methylation with increase in age as compared to controls [32]. Tai Chi practitioners also have a lower amount of severely damaged DNA along with stronger DNA repair mechanisms, advanced levels of lymphocyte renewal, and active endogenous antioxidant enzymes that reduce oxidative damage [122, 123]. It is possible that such slowing of age-related changes in methylation may protect against inevitable deterioration of epigenetic regulatory mechanisms that occur as a function of age [124]. A possible link between Tai Chi and antiaging effects might involve epigenetic regulation of progenitor cell proliferation. Notably, Tai Chi practitioners were observed to have an increase in the levels of CD34+ progenitors in peripheral blood that correlated with promotion of regenerative health [125].

#### 3.2.12. Qi Gong

The practice of Qi Gong follows a combination of movement, self-massage, meditation, and breathing where mindfulness is crucial for attaining heightened consciousness. Qi Gong is associated with an increase in antioxidant activity, measured in terms of urine malondialdehyde and superoxide dismutase [126]. Qi Gong as a superior alternative to other antiaging exercises is endorsed by the fact that it may prevent conditions such as hypertension, cardiovascular disease, asthma, allergies, neuromuscular problems, and cancer through epigenetic mechanisms [127].

### 3.3. Other Complementary Health Approaches

All other practices of treating illness by nonconventional, traditional methods that have not been included in the two previous categories have been categorized by the NCCIH as other complementary health approaches.

#### 3.3.1. Ayurvedic Medicine

Ayurveda is an ancient Indian discipline based on a personalized approach to medicine. The practice was passed down orally for thousands of years. This wisdom is corroborated with rare original texts such as the Charaka Samhita (poetic verses outlining internal medicine) and the Sushruta Samhita (a similar work focused on Ayurvedic surgery) dating from the dawn of the current era. The very term “Ayurveda” derives from the classical Sanskrit words “ayu” meaning life and “veda” meaning knowledge or science. This venerable practice can be used to classify everyone into unique prakritis (representing the nature of an individual's mind and body constitution) based on the theory of tridosha (the three major constitutional types). Tridoshas, namely, vata (principles of motion), pitta (metabolism), and kapaha (structure), are phenotypic psychophysiological principles and groupings based on the nature of energies derived from a combination of the five elements (space, air, water, fire, and earth) and their related properties. Tridoshas account for mental, morphological, and metabolic (body) characteristics of the human body. A unique combination of the three different doshas, vata, pitta, and kapha, characterizes each individual and endows them with a unique prakriti type. Furthermore, prakriti-based Ayurvedic medicine is based on prakriti-specific genomes, constituting an additional layer of unique personalized medicine [128]. Additional studies are required to develop a sound understanding of potency, selectivity, and side effects at play in Ayurvedic practice and allow selection of the optimal type of IM practice necessary for achieving wellness.

Ayurveda also defines comprehensive personality types and traits based on gunas (qualities of the mind and consciousness). Prakritis in addition to the tridoshas (bodily traits) are influenced by a combination of trigunas (three types of psychological traits), namely, sattva (intelligence, happiness), rajas (energy such as pain causing imbalance), and tamas (substance, inertia) [129].

The dominance of gunas defines a person's temperament, and this plays a major role in daily activities and diet [129]. Such psychological traits (gunas) might be intertwined with physical characteristics (doshas) to contribute to lifestyle and behavior and could be used as the deterministic factor in selecting the type of Ayurvedic medicine used to treat a malady.

This type of individualized classification may provide phenotypic datasets suitable for analysis of underlying genetic and epigenetic variation and in addition may offer promising insight necessary for making truly personalized medicine a reality. The prognosis, diagnosis, and therapeutics in Ayurvedic practice are prakriti-specific and have similarities with modern concepts of pharmacogenomics. Ayurvedic medicine focuses on diet (ahara), lifestyle (vihara), and medication (aushadhi) in a vital and holistic approach to health. With an emphasis on prevention of disease, Ayurvedic medicine offers promising evidence in treating and managing chronic ailments [130].

Single nucleotide polymorphisms (SNPs) and epigenetic factors capable of influencing drug response currently represent the cutting edge of personalized medicine from a Western perspective, but in the Eastern tradition of Ayurveda one drug does not fit all. Recent findings have identified a correlation between HLA alleles and prakriti type (akin to phenotype), establishing a rationale supporting the Ayurvedic tridosha approach to personalized medical treatment based on individual prakriti types used as biomarkers for genotype/phenotype [131]. A broad analysis of genome-wide SNPs in the Indian male population has revealed that specific SNPs corresponded to specific prakritis [128]. Furthermore, to correlate the functional relevance of SNPs and associated genes, the study found that the gene PGM1, involved in several metabolic pathways such as glycolysis, correlates with the phenotype of pitta (characteristically involving digestion, metabolism, and energy production), demonstrating the genetic validity of Ayurvedic prakriti classification [128].

The fact that Ayurvedic theory has emphasized personalized health care for over 5,000 years is notable. Perhaps more amazing is the fact that it has long recognized the differences in individual metabolic rates; for example, individuals with pitta prakriti are fast metabolizers while those with kapha prakriti are slow metabolizers [130]. It is known that metabolic CYP enzymes show correlations between individual metabolic polymorphisms and prakriti type [132]. In a cohort of patients with rheumatoid arthritis, 21 markers were evaluated to assess putative correlations between genes prevalent in inflammatory and oxidative stress pathways and prakriti type. Findings revealed that SNPs in inflammatory gene markers for IL1*β*, TNF*α*, and CD40 were determinants for individuals in the vata subgroup, whereas those for oxidative stress gene markers for SOD3 and PON1 were stronger determinants for individuals in the pitta subgroup, and only negligible associations of SNPs for SOD3 and PTPN22 gene markers were correlated with individuals in the kapha subgroup [133].

From an epigenetic perspective, a detailed microarray analysis of DNA methylation across prakriti phenotypes using methylated DNA immunoprecipitation (MeDIP) revealed prakriti-specific signatures of CpG islands and promoters/UTRs amongst pitta prakriti, vata prakriti, and kapha prakriti. This confirms an epigenetic basis for the underlying prakriti classification [134]. Such epigenetic signatures corresponding to individual prakritis may be crucial in understanding and harnessing the power of Ayurvedic medicine and ascertaining its mechanism for delivering beneficial results in health and wellness. Data such as these provide a molecular framework upon which to compare the holistic principles of Ayurvedic medicine to genetic variation and epigenetic regulation that underlie differential gene expression and predisposition to disease.

Ayurvedic science also advocates the use of herbs and spices in addition to foods for their medicinal properties, and epigenetic properties have recently been identified in many of these comestibles. For example, the neuroprotective, neurotrophic, and anti-inflammatory properties of ginger can be attributed to [6]-shogaol, the bioactive component that regulates histone H3 acetylation, suppresses histone deacetylase (HDAC)1 expression, and increases the expression of HSP70 that leads to improvement in neurological performance [135]. Other foods with a long Ayurvedic tradition and capable of epigenetic regulation include honey, saffron, and ghee. With further study, dietary chemoprevention using phytochemicals and other tools of Ayurvedic medicine may one day bridge the gap between molecular alterations under epigenetic control and comprehensive treatment of discomfort, imbalance, and neoplasia [136].

#### 3.3.2. Homeopathy

Although certainly controversial, homeopathy is a system of medicine that employs a single, focused approach to treatment. Philosophically, it is based on the “law of similars” (what a substance can cause, it can cure). Homeopathic medicine also places great importance on the evaluation and consideration of ancestral health conditions while shortlisting remedies for treating the patient. This approach maintains that ancestral imprints are carried transgenerationally; thus, homeopathy strives to reverse the negative imprint and silence it permanently by influencing the immune system. Epigenetic theory suggests that unique diet, health, and stress patterns from parents can also be passed on transgenerationally. Therefore, homeopathic medicine may be working epigenetically to reverse diseased conditions such that the results are long lasting, even in terms of curing congenital weaknesses.

Global genomic hypomethylation and a dense hypermethylation of the CpG islands associated with gene regulatory regions are a hallmark of cancer cells that are often caused by environmental factors [137]. In various instances, homeopathic medicines have shown their potential as chemotherapeutic remedies and their mechanism of action involves the reversal of the epigenetic signature unique to cancer cells. For example, the homeopathic drug* Lycopodium clavatum* has been observed to cause cell death by induction of apoptotic gene expression in HeLa cells without any detectable cytotoxic effect on normal PBMCs, suggesting its possible use as a safe anticancer drug [138]. Another set of homeopathic drugs, Condurango and Hydrastis Canadensis, when administered to HeLa cells displayed a pattern of over 100 differentially expressed genes suggesting the power of ultra-diluted homeopathic medicines in reversing aberrant epigenetic cancer signatures [139]. Condurango-treated lung cancer cells have shown a significant decrease in hypermethylation patterns associated with tumor suppressor genes such as p15 and p53 in vitro, as well as inhibition of hypermethylation of p15 in post-cancer treatment of rat models [140].

#### 3.3.3. Naturopathy

Naturopathic medicine is a system for treating disease using a broader approach than homeopathy. It strives to determine the cause of a patient's complaint and an environment that supports recovery and to this end relies on natural therapies utilizing nutrition, herbs, iridology, massage, and homeopathic treatments to achieve balance. Just like Ayurveda, naturopathy is a mind-body medicine that promotes self-healing. Naturopathic treatments rely and focus on the six maxims (principles): the healing power of nature, identify and treat the cause, treat the whole person, first do no harm, doctor as teacher, and last but not least prevention [141]. Naturopathists frequently employ multiple prescriptions per treatment and typically rely on use of natural agents such as supplements and herbs plus noninvasive physical techniques in lieu of drugs and invasive procedures.

Propolis, a honey-bee derived naturopathic formulation, contains caffeic acid phenethyl ester (CAPE) as a core component and has been shown to induce cellular differentiation, cell cycle arrest, and apoptosis in breast cancer cell lines. CAPE acts as an HDAC inhibitor resulting in accumulation of acetylated histone proteins in MCF-7 and MDA-231 breast cancer cell lines, resulting in downregulation of estrogen and progesterone receptors in MCF-7 cell lines. This leads to cell differentiation and inhibition of the mdr-1 gene which would otherwise confer resistance to chemotherapeutic drugs in cancer cells [142]. In addition, n-3 polyunsaturated fatty acids, found in marine fish oils, have been shown to enhance immunity in obese mice by elevating the levels of B cells and circulating IgM in vivo [143], possibly indicative of a similar epigenetic mechanism.

### 3.4. The Placebo Effect

Placebo treatments are innocuous procedures carried out in clinical trial settings that are known to have beneficial health effects on healthy volunteers as well as patients [144]. Placebo effects operate through an underlying psycho- and/or neurobiological mechanism. One such mechanism is based on the effects of dopamine, which is known as an integrator of placebo response. The availability of dopamine at synapses is responsible for several effects pertaining to reward-motivated behavior. Dopamine clearance at synapses is carried out by catechol-O-methyltransferase (COMT). One study demonstrates that people suffering from IBS with a COMT val158met polymorphism exhibited improved symptomatology following administration of placebo treatment alone [145]. This exemplifies how genetics can modify the response to a placebo, which by itself may not represent an actual therapeutic or alleged therapeutic practice. Whether any of the CAM practices intensify such effects needs to be extensively investigated and accounted for in case studies.

## 4. Conclusions

Integrative Medicine represents a field that attempts to integrate time-honored healing practices employing environmental, body, mind, consciousness, and more esoteric elements to modern medical tools and applications. This unique and powerful holistic approach is gathering momentum as much for its clinical relevance as for its potential applications in nontraditional health care, behavior, and lifestyle [146, 147]. Currently, more than one third of American adults partake of IM in one form or another [33]. Progressive thinkers in science and medicine are highly encouraged by the whole-systems philosophy of IM and are eager to identify the mechanisms underlying its benefits [22]. Based on numerous findings, we advocate wider acceptance and application of these techniques, but with careful discernment and bearing in mind their limitations when used instead of conventional medicine for treating serious conditions. The recent shift in Western medicine from the limited, one-dimensional focus on acute-phase treatments to a more expansive and comprehensive gestalt gives equal attention to chronic illness and lifestyle-related morbidity. Coupled with a growing aging population, these shifting perspectives combine to forge an atmosphere conducive to embracing IM and supporting continued research into its mechanisms [148].

The idea that IM possesses the power to regulate the epigenome to achieve its therapeutic effects is novel and needs further development and could play a crucial role in developing future research strategies in health care. Furthermore, the methodical analysis of IM-induced epigenetic modulations described herein demonstrates the necessity for an integrated approach to treating ailments and diseases that are currently impervious to the conventional medical arsenal. Furthermore, research efforts must continue to focus on the healing modalities of IM to best interpret the roles that epigenetic regulation, gene expression, and other relevant biomarkers play in the healing process. By elucidating these mechanisms, the therapeutic fidelity of medical treatment, including IM, will improve and pave the way for future disease prevention, treatment, and eradication.

Traditional forms of CAM have been successfully practiced for millennia, but a molecular approach to medicine now prevails. Whether acting through material, temporal, energetic, or spatial dimensions, we propose that revered ancient IM techniques operate, at least in part, through molecular regulation, specifically, epigenetic modification, to achieve their healing function. Identification of this epigenetic connection between IM and gene expression makes it possible for humans not merely to heal, but to thrive. This is especially true with respect to temporal and energy medicine, where mind over matter becomes more than a catchy phrase; it could literally be “mind over gene” in this context. Our work describes how IM may function as an epigenetic modulator for equilibrating the body to peak efficiency and wellness. Furthermore, continued investigation into the molecular mechanisms responsible for the healing effects conveyed through IM will generate valuable insight into the role of epigenetics in healing and contribute to improvements in overall treatment outcome, wellbeing, and longevity.

We have attempted to assimilate the current knowledge of different types of CAM approaches that together represent the whole-systems wisdom inherent to IM that have to date rarely been compared, dissected, or compiled and combined it with our understanding of epigenetics. The fact that these practices originated in different geographical locations around the globe at different time points in history highlights their strong cultural associations and uniqueness. However, we believe that these approaches collectively share a common basic mechanistic paradigm, that is, modification of the epigenetic landscape. This is supported by the increasing number of studies suggesting epigenetics as the converging mechanism of all IM products and practices. To strengthen our point, we also highlight similarities and differences between different practices that originated in the same or different cultures. For example, both yoga and acupuncture are mind-body practices that originated in different geographical civilizations but operate through different elements; the mechanistic mode of action of yoga is via spatial rearrangement of body whereas acupuncture is via channeling of energy. However, they still converge at the basic mechanistic paradigm that is epigenetic modification. Such characterizations can bring us one step closer to realizing the potential of these ancient popular practices that have gained popularity in recent years and are routinely being incorporated into Western culture. In addition to the emerging field of research in IM supported by the NCCIH, it will be beneficial to look at quantitative and qualitative epigenetic changes resulting from individual and/or combinations of IM practices to further test their efficacy and safety, as well as to improvise on already existing therapeutic strategies to prevent or cure disease and disability.

## Abbreviations

IM: Integrative Medicine
CAM: Complementary and alternative medicine
TCM: Traditional Chinese Medicine
ANS: Autonomic nervous system
CH3: Methyl group
DNMT: DNA methyltransferase
HDAC: Histone deacetylase
HAT: Histone acetyltransferase
HMT: Histone methyltransferase
HDM: Histone demethylase
SAMe: S-Adenosyl methionine
HM: Herbal methionine
MKP-1: Mitogen-activated protein kinase phosphatase 1
ERK: Extracellular-signal Regulated Kinase
SNPs: Single nucleotide polymorphisms
MeDIP: Methylated DNA immunoprecipitation
PTSD: Posttraumatic stress disorder
MI: Myocardial infarction
VEGF: Vascular endothelial growth factor
LG: Licking or grooming
COMT: Catechol-O-methyltransferase.
